# Answering complex hierarchy questions in network meta-analysis

**DOI:** 10.1186/s12874-021-01488-3

**Published:** 2022-02-17

**Authors:** Theodoros Papakonstantinou, Georgia Salanti, Dimitris Mavridis, Gerta Rücker, Guido Schwarzer, Adriani Nikolakopoulou

**Affiliations:** 1 Institute of Medical Biometry and Statistics, Faculty of Medicine and Medical Centre-University of Freiburg, Freiburg, Germany; 2 Institute of Social and Preventive Medicine (ISPM), University of Bern, Bern, Switzerland; 3 Department of Primary Education, University of Ioannina, Ioannina, Greece; 4 Faculté de Médecine, Université Paris Descartes, Paris, France

**Keywords:** Clinically relevant question, Indirect evidence, Probabilistic ranking, Evidence synthesis

## Abstract

**Background:**

Network meta-analysis estimates all relative effects between competing treatments and can produce a treatment hierarchy from the most to the least desirable option according to a health outcome. While about half of the published network meta-analyses present such a hierarchy, it is rarely the case that it is related to a clinically relevant decision question.

**Methods:**

We first define treatment hierarchy and treatment ranking in a network meta-analysis and suggest a simulation method to estimate the probability of each possible hierarchy to occur. We then propose a stepwise approach to express clinically relevant decision questions as hierarchy questions and quantify the uncertainty of the criteria that constitute them. The steps of the approach are summarized as follows: a) a question of clinical relevance is defined, b) the hierarchies that satisfy the defined question are collected and c) the frequencies of the respective hierarchies are added; the resulted sum expresses the certainty of the defined set of criteria to hold. We then show how the frequencies of all possible hierarchies relate to common ranking metrics.

**Results:**

We exemplify the method and its implementation using two networks. The first is a network of four treatments for chronic obstructive pulmonary disease where the most probable hierarchy has a frequency of 28%. The second is a network of 18 antidepressants, among which Vortioxetine, Bupropion and Escitalopram occupy the first three ranks with frequency 19%.

**Conclusions:**

The developed method offers a generalised approach of producing treatment hierarchies in network meta-analysis, which moves towards attaching treatment ranking to a clear decision question, relevant to all or a subset of competing treatments.

## Background

Providing a treatment hierarchy is often one of the objectives of systematic reviews that contain multiple interventions [1]. To this aim, several ranking metrics have been developed and are commonly used to accompany network meta-analysis (NMA) results [2]. A common output of NMA is a matrix showing the probability of each treatment being at each possible rank. The graphical display of such a matrix is called rankogram, the restriction of this matrix to the probabilities of occupying the highest rank constitutes the probability of being the best ranking metric, while the Surface Under the Cumulative RAnking curve (SUCRA) summarises the ranking distribution by calculating the area under the cumulative ranking curve [3]. Mean and median ranks are further options to present a treatment hierarchy, with the former being a linear transformation of SUCRA. The P-score measure bypasses the need to calculate probabilities of being at each rank by averaging over probabilities of each treatment being better than any other in the network [4]. P-scores are the frequentist analogue of SUCRAs given that the posterior distributions of relative effects are normal. P-scores have recently been adapted to the Bayesian framework and extended to the predictive P-scores for a future study setting [5].

Despite the plethora of ranking metrics and the popularity of deriving a treatment hierarchy in NMA, relying on such a treatment hierarchy is insufficient. Limitations of ranking treatments include the fact that small differences in outcome values could lead to different hierarchies even if these differences are not clinically relevant, the difficulty in interpreting the values of the ranking metrics and the lack of consideration of multiple outcomes [6, 7]. This has led to the development of several multi-dimensional approaches to treatment ranking including benefit-risk assessments [8–10], incorporation of clinically important values [3, 10, 11], consideration of multiple outcomes simultaneously [7, 10, 12] or consideration of a characteristic such as risk of bias when deriving a treatment hierarchy [13]. Moreover, Salanti et al. recently proposed that each ranking metric answers a different treatment hierarchy question [14], and thus differences in the produced hierarchies are to be expected [15]. Linking the ranking metrics with the respective hierarchy question they answer would greatly facilitate the interpretation of the derived hierarchy.

However, the hierarchy questions of interest are not limited to those that can be answered by the available ranking metrics. Often, more complex research questions are posed; in such a case, hierarchy should still depend on these questions, so that its interpretation is relevant and meaningful. In this paper, we suggest an approach for translating clinically relevant questions into hierarchy questions and quantify their uncertainty. To this aim, we use simulations to derive the relative frequency of all possible hierarchies in a network of interventions. Then, we define the set of all possible hierarchies that satisfy a specified criterion, for example that a specific order among treatments is retained in the network and/or a treatment is in a specific position, and the sum of their frequencies constitute the certainty around the criterion.

## Methods

### Definitions: treatment hierarchy and treatment ranking

Let the entire evidence base form a set $\mathbbm{T}=\left\{{t}_1,{t}_2,\dots, {t}_T\right\}$ (ordered alphabetically) of *T* treatments. NMA aims to estimate the set of $$\left(\begin{array}{c}T\\ {}2\end{array}\right)$$ relative treatment effects ${\mu}_{t_i{t}_j}$ where *t*_*i*_*t*_*j*_ denotes the treatment contrast (*i*, *j* = 1, …, *T*; *i* < *j*) [16, 17]. The parameters ${\mu}_{t_i{t}_j}$ denote additive effects, e.g. mean differences or log-odds ratios, where ${\mu}_{t_i{t}_i}=0$. The model is parametrized using only *T* − 1 relative treatment effects versus a randomly selected reference treatment, here *t*_1_. The so called ‘basic parameters’ ${\mu}_{t_1{t}_i}$ are estimated and collected in a vector $\hat{\boldsymbol{\mu}}$ and we denote their variance-covariance matrix as $\hat{\boldsymbol{V}}$. The remaining $$\left(\begin{array}{c}T\\ {}2\end{array}\right)-T+1=$$ $$\left(\begin{array}{c}T-1\\ {}2\end{array}\right)$$ relative treatment effects are derived imposing the constraint of consistency ${\mu}_{t_i{t}_j}={\mu}_{t_k{t}_j}-{\mu}_{t_k{t}_i}$, *k* ≠ *i*, *j*, *k* = 1, …, *T* [18, 19].

The ‘true’ underlying *treatment hierarchy* for the set $\mathbbm{T}$ is defined as the vector of treatment names, ordered from the most to the least effective. This hierarchy is imposed by the ascending ordering of the ‘true’ underlying relative treatment effects, ${\mu}_{t_1{t}_i}$, assuming a direction, e.g. that large positive values are associated with a beneficial effect for the first treatment. The ‘true’ underlying *treatment ranking* is defined as the vector of integers between 1 and *T* that indicates the rank of each treatment ${r}_{t_i}$. For example, if the treatment hierarchy vector is (A, C, D, B), then the treatment ranking vector is (1, 4, 2, 3). We denote the ‘true’ underlying treatment ranking as

$$\mathbf{R}:= \left({r}_{t_1},{r}_{t_2},\dots, {r}_{t_T}\right)$$

which has a 1:1 correspondence with the ‘true’ underlying treatment hierarchy

$$\mathbf{H}:= \mathbbm{T}\ ordered\ by\ \left({r}_{t_1},{r}_{t_2},\dots, {r}_{t_T}\right).$$

**Fig. 1:**
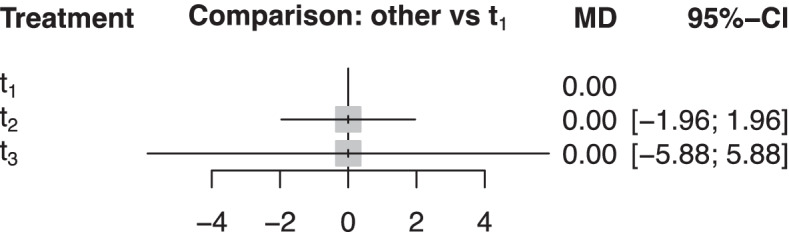
Network meta-analysis relative treatment effects for a hypothetical network of three treatments, *t*_1_, *t*_2_ and *t*_3_. MD: mean difference; CI: confidence interval

The estimated distribution of **R** is denoted as $\hat{R_{\mathbbm{T}}}$ and is approximated from the estimated relative treatment effects ${\hat{\mu}}_{t_i{t}_j}$ as follows. First, we sample from the multivariate normal distribution with point estimate $\hat{\boldsymbol{\mu}}$ as mean and variance-covariance matrix $\hat{\mathbf{V}}$. In the case of a Bayesian analysis, we do not need to assume a normal approximation, but the whole posterior distribution of ***μ*** can be considered. Then, from the approximated normal distribution or the posterior, we can draw a ***μ***^***∗***^ vector and get corresponding ranks $\left({r_{t_1}}^{\ast },{r_{t_2}}^{\ast },\dots, {r_{t_T}}^{\ast}\right)$. Repeating the process a large number of times, say *n*, will produce a matrix of dimension *n* × *T*, which is a sample from the distribution $\hat{R_{\mathbbm{T}}}$. Then, by using the 1:1 correspondence with the treatment names, we can produce a sample from $\hat{H_{\mathbbm{T}}}$, which is the estimated distribution of **H**. The larger the number of treatments included in a network, the greater *n* should be. A theoretical example of samples from $\hat{R_{\mathbbm{T}}}$ and $\hat{H_{\mathbbm{T}}}$ is presented in Table 1 panel a for a hypothetical network of three treatments, results of which are shown in Fig. 1.

**Table 1:** Sample from $\hat{R_{\mathbbm{T}}\ }$ and $\hat{H_{\mathbbm{T}}\ }$ (panel a), ranking matrix (panel b) and hierarchy matrix (panel c) of the hypothetical network of three treatments *t*_1_, *t*_2_ and *t*_3_ of Fig. 1

Panel a						Panel b Ranking matrix: Summary of the $\hat{H}_{T}$ sample to show uncertainty in the ranking of each treatment				Panel c Hierarchy matrix: Estimated probability mass function of treatment hierarchy	
Sample from $\hat{{R}_{T}}$			Sample from $\hat{{H}_{T}}$								
${{r}_{{t}_{1}}}^{\ast}={3}$	${{r}_{{t}_{2}}}^{\ast}={2}$	${{r}_{{t}_{3}}}^{\ast}={1}$	*t*_3_	*t*_2_	*t*_1_		1st	2nd	3rd	*h*_*l*_	${p}_{{h}_{l}}$
${{r}_{{t}_{1}}}^{\ast}={2}$	${{r}_{{t}_{2}}}^{\ast}={1}$	${{r}_{{t}_{3}}}^{\ast}={3}$	*t*_2_	*t*_1_	*t*_3_	*t*_1_	25%	50%	25%	{*t*_3_, *t*_1_, *t*_2_} (*h*_1_)	25%
${{r}_{{t}_{1}}}^{\ast}={1}$	${{r}_{{t}_{3}}}^{\ast}={2}$	${{r}_{{t}_{2}}}^{\ast}={3}$	*t*_1_	*t*_3_	*t*_2_	*t*_2_	30%	40%	30%	{*t*_2_, *t*_1_, *t*_3_} (*h*_2_)	25%
${{r}_{{t}_{1}}}^{\ast}={2}$	${{r}_{{t}_{2}}}^{\ast}={3}$	${{r}_{{t}_{3}}}^{\ast}={1}$	*t*_3_	*t*_1_	*t*_2_	*t*_3_	45%	10%	45%	{*t*_3_, *t*_2_, *t*_1_} (*h*_3_)	20%
${{r}_{{t}_{1}}}^{\ast}={1}$	${{r}_{{t}_{2}}}^{\ast}={2}$	${{r}_{{t}_{3}}}^{\ast}={3}$	*t*_1_	*t*_2_	*t*_3_					{*t*_1_, *t*_2_, *t*_3_} (*h*_4_)	20%
${{r}_{{t}_{1}}}^{\ast}={2}$	${{r}_{{t}_{2}}}^{\ast}={1}$	${{r}_{{t}_{3}}}^{\ast}={3}$	*t*_2_	*t*_1_	*t*_3_					{*t*_2_, *t*_3_, *t*_1_} (*h*_5_)	5%
${{r}_{{t}_{1}}}^{\ast}={2}$	${{r}_{{t}_{2}}}^{\ast}={3}$	${{r}_{{t}_{3}}}^{\ast}={1}$	*t*_3_	*t*_1_	*t*_2_					{*t*_1_, *t*_3_, *t*_2_} (*h*_6_)	5%
${{r}_{{t}_{1}}}^{\ast}={1}$	${{r}_{{t}_{2}}}^{\ast}={2}$	${{r}_{{t}_{3}}}^{\ast}={3}$	*t*_1_	*t*_2_	*t*_3_						
${{r}_{{t}_{1}}}^{\ast}={2}$	${{r}_{{t}_{2}}}^{\ast}={1}$	${{r}_{{t}_{3}}}^{\ast}={3}$	*t*_2_	*t*_1_	*t*_3_						
${{r}_{{t}_{1}}}^{\ast}={3}$	${{r}_{{t}_{2}}}^{\ast}={2}$	${{r}_{{t}_{3}}}^{\ast}={1}$	*t*_3_	*t*_2_	*t*_1_						
			…								
			…								
			…

The sample from $\hat{R_{\mathbbm{T}}}$ is summarized in what we will call the *ranking matrix*, in which each entry ${p}_{t_i,r}=P\left({\hat{r}}_{t_i}=r| data\right),$ *r* = 1, …, *T* shows the proportion of times (the frequency) each treatment *t*_*i*_ being at each possible rank *r*. The estimated probability that treatment *t*_*i*_ occupies the *r*th rank means that it produces better values than exactly *T* − *r* treatments. These probabilities have been presented in the literature in graphs called rankograms. Table 1 panel b shows the ranking matrix for the hypothetical network of Fig. 1.

The *T*! possible treatment hierarchies in a network of *T* treatments are denoted as *h*_*l*_, *l* = 1, …, *T*!. The probability mass function of *h*_*l*_ is derived by summarizing the sample from $\hat{H_{\mathbbm{T}}}$ in a matrix that we call the *hierarchy matrix*. A particular hierarchy *h*_*l*_ features *x* times in the sample and this defines as ${p}_{h_l}=x/n$ the relative frequency that this hierarchy occurs. The hierarchy matrix is ordered by decreasing frequency that a hierarchy occurs, i.e., *h*_1_ corresponds to the most frequent hierarchy. It is also possible that more than one most probable hierarchy exists in a network due to ties. As is the case with ${p}_{t_i,r}$, the estimated probability of each possible hierarchy ${p}_{h_l}$ depends on the data. For small *n*, several hierarchies will have an estimated probability of 0. Table 1 panel c shows the hierarchy matrix for the example in Fig. 1.

### An approach for answering clinically relevant decision questions

In the following, we propose a stepwise approach to express clinically relevant decision questions as hierarchy questions and quantify the uncertainty of the criteria that constitute them. We have developed an R package **nmarank**, hosted in CRAN [20], which allows users to implement the suggested approach [21]. In [22] readers can find the current version of the **nmarank** package which can be used to reproduce the results presented in the manuscript. The documentation of the package version 0.2–3 serves as a guide of the functions included in the **nmarank** package. The suggested approach is outlined below.

#### Step 1: define a clinically relevant research question

In the first step of the approach, investigators set a question that is considered clinically important. Different users of NMA would normally consider different questions to be of clinical importance; e.g. clinicians might be interested in general questions that capture the entire population of patients, policy decision makers might ask specialized questions while patients might seek answers to research questions focused on their specific patient group. Also, questions may include all treatments in the network or focus on a subset of them, as it is often the case in a clinical setting.

For example, a question of clinical relevance to a decision maker might be whether a treatment *t*_*i*_ has better outcome than another (possibly effective but more expensive) treatment *t*_*j*_. Alternatively, clinicians might be interested to know the top three treatments in the network. Other examples of questions include that a specific order *t*_*i*_, *t*_*j*_, *t*_*k*_ is retained anywhere in the hierarchy, that a treatment *t*_*i*_ occupies a specific rank *r* or that it is among the best two ranks. It is also possible that we are interested in the case that a treatment *t*_*i*_ has better outcome than treatment *t*_*j*_, but against a clinically important value c $\left({\mu}_{t_i{t}_j}>\mathrm{c}\right)$ instead of their differences being zero $\left({\mu}_{t_i{t}_j}>0\right)$. Depending on the context, it might also be the case that a combination of criteria constitutes a clinically relevant question. As an example, we might be interested in the case where *t*_*i*_ is first or second and *t*_*j*_ has better mean outcome value than that of treatment *t*_*k*_ plus a clinically important value c = 0.5. As a special case of a clinically relevant question could be that a specific treatment hierarchy occurs, for example that imposed by the estimated relative treatment effects, expressed as *t*_*i*_ is first, *t*_*j*_ is second, *t*_*k*_ is third and so on.

#### Step 2: find hierarchies compatible with the defined question

After setting the decision question of interest, the aim is to define the set of possible hierarchies out of all *T*! hierarchies that satisfy the criteria set in **Step 1**. This is done by selecting those hierarchies in the sample of $\hat{H_{\mathbbm{T}}}$ for which the criterion is satisfied, thus translating the decision question into a hierarchy question. Depending on the context, the selected hierarchies might be of interest on their own or might only be used for proceeding to **Step 3**.

If we are interested in a question involving a clinically important value c, then the respective criteria need to be applied to mark the hierarchies that satisfy them in the sampling process of $\hat{R_{\mathbbm{T}}}$ and $\hat{H_{\mathbbm{T}}}$ described in section Definitions: treatment hierarchy and treatment ranking. For example, if we are interested in the combination of criteria that *t*_*i*_ is first or second and *t*_*j*_ has better mean outcome value than that of treatment *t*_*k*_ plus a clinically important value c = 0.5, then we need to differentiate between the two types of hierarchies: those where in the sampling the condition ${\mu}_{t_j{t}_k}>\mathrm{c}$ will be satisfied and those where it will not.

#### Step 3: define certainty that the criterion is satisfied

In **Step 3** of the framework, we add the frequencies of the hierarchies ${p}_{h_l}$ selected in **Step 2** that satisfy the decision criterion set in **Step 1**. In our example from Fig. 1, the estimated probability that treatment *t*_1_ is at higher rank as *t*_2_ is the sum of the frequencies of *h*_1_, *h*_4_ and *h*_6_ hierarchies which amounts to 50% (see Table 1). Considering a case where we combine two criteria, say that we are interested in the case where *t*_1_ is first or second and *t*_2_ is higher in the hierarchy than *t*_3_. Then, we add the frequencies of *h*_2_ and *h*_4_ hierarchies, which amounts to ${p}_{h_2}+{p}_{h_4}=45\%.$ It might be the case that not all *T*! possible hierarchies are included in the sample of $\hat{H_{\mathbbm{T}}}$; this, however, does not pose a problem in the process as the most frequent ones are recorded and used to estimate the certainty of the criterion.

#### Evaluation of certainty of the criterion

As an extra step of the approach, the amount of certainty of the criterion can be evaluated. This is done by comparing the frequencies derived in **Step 3** with the respective frequencies corresponding to other relevant questions, in a similar manner that Bayes factors are derived [23]. For example, we may want to compare the estimated probability that three particular treatments from a family of interventions occupy the first three ranks, *p*_*A*_, versus the respective probability that three other treatments from another family of interventions do, *p*_*B*_, and we do so by taking their ratio $\frac{p_A}{p_B}$. Alternatively, in a similar setting where we are interested in the optimal family of treatments, consider that *t*_*i*_ and *t*_*k*_ are the best candidates for family A and *t*_*j*_ and *t*_*m*_ are the best candidates for family B, *m* ≠ *i*, *j*, *k*, *m* = 1, …, *T*. Then, we may compare the frequencies that *t*_*i*_ has better mean outcome value than *t*_*j*_ and *t*_*k*_ has better mean outcome value than treatment *t*_*m*_ versus the probability that *t*_*j*_ has better mean outcome value than *t*_*i*_ and *t*_*m*_ has better mean outcome value than treatment *t*_*k*_.

### Relation of the hierarchy matrix with common ranking metrics

#### Estimated NMA relative treatment effects

The estimated ${H}_{\mathbbm{T}}$ and ${R}_{\mathbbm{T}}$ are *T*-dimensional distributions constructed from the estimated multivariate normal distribution of the relative treatment effects. A point estimate of the distribution $\hat{H_{\mathbbm{T}}}$ can be obtained by ordering the point estimates of ${\hat{\mu}}_{t_1{t}_i}$ for each ${t}_i\in \mathbbm{T}$. This estimated treatment hierarchy might or might not be the same as the mode of $\hat{H_{\mathbbm{T}}}$, which is the hierarchy *h*_1_ (the most probable hierarchy). Below we show a hypothetical example with three treatments, *t*_1_, *t*_2_, *t*_3_, of a network with three treatments where *h*_1_ differs from the hierarchy imposed by the NMA estimated relative treatment effects.

**Fig. 2:**
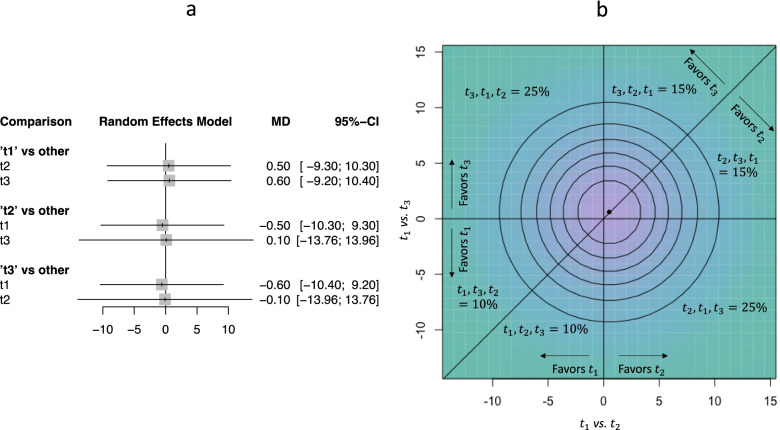
Network meta-analysis relative treatment effects of a hypothetical network of three treatments, *t*_1_, *t*_2_ and *t*_3_ (panel **a**) and two-dimensional plot of the probability density function for the bivariate normal distribution corresponding to the network meta-analysis of panel a with the associated frequencies of all possible hierarchies (panel **b**). MD: mean difference

Figure 2 panel a shows the relative treatment effects of all treatments versus each other. The hierarchy that occurs from the resulted mean differences is {*t*_3_, *t*_2_, *t*_1_}. Fig. 2 panel b illustrates in a two-dimensional plot the probability density function for the bivariate normal distribution $\hat{\boldsymbol{\mu}}=\left({\hat{\mu}}_{t_1{t}_2},{\hat{\mu}}_{t_1{t}_3}\right)=\left(\mathrm{0.5,0.6}\right)$ with variance-covariance matrix $$\hat{\mathbf{V}}=\left(\begin{array}{cc}25& 0\\ {}0& 25\end{array}\right)$$. The *T* !  = 6 possible hierarchies are represented by the regions separated by the straight lines in Fig.2 panel b; each of the three straight lines divides the area according to whether each pairwise comparison favors the one or the other treatment. The region corresponding to {*t*_3_, *t*_2_, *t*_1_} includes the point estimate (0.5,0.6), noted as the black dot. However, this is not the most probable hierarchy (frequency 15%) as the probability mass is smaller than for other regions; hierarchies {*t*_3_, *t*_1_, *t*_2_} and {*t*_2_, *t*_1_, *t*_3_} are more probable than that of the mean effects, each one having 25% probability of occurring.

#### Probability of producing the best value

The first column of the ranking matrix shows the frequency that each treatment occupies the highest rank and has been frequently used as a ranking metric usually referred as “probability of being best”. “Probability of being best”, however, has been recently indicated as an inaccurate name for the particular ranking metric as “being the best” may have a large variety of meanings and interpretations [14]. “Probability of producing the best value” has been suggested instead as better reflecting the nature of the particular ranking metric and this name is also adopted in this paper.

The hypothetical triangular network of Fig. 1 is sometimes used to criticize the probability of producing the best value ranking metric. It holds that ${\hat{\mu}}_{t_1{t}_2}={\hat{\mu}}_{t_1{t}_3}=0$, rendering each treatment to have 50% probability of being better than any other, but the former relative effect is associated with greater precision than the later. In such cases, probability of producing the best value favours treatments estimated with greater uncertainty. As indicated in Table 1 panel b (and can be also easily derived from Table 1 panel c), treatment *t*_3_ has a probability of producing the best value 45%, followed by *t*_2_ with a probability of 30%, while *t*_1_ is associated with 25% probability of producing the best value. However, hierarchy {*t*_3_, *t*_1_, *t*_2_} is equally probable with {*t*_2_, *t*_1_, *t*_3_} (25%) and hierarchies {*t*_3_, *t*_2_, *t*_1_} and {*t*_1_, *t*_2_, *t*_3_} are also equally probable (20%). The hierarchy matrix highlights that given that one of the three treatments is second, the other two have equal estimated probabilities of being first or third. The large tails of the distributions of relative treatment effects of *t*_3_ versus the other two treatments make it improbable that it ranks on the second position, rendering hierarchies {*t*_2_, *t*_3_, *t*_1_} and {*t*_1_, *t*_3_, *t*_2_} occurring each in 5% of the simulations.

#### SUCRAs and P-scores

As shown in the example of section Probability of producing the best value, the probability of producing the best value can be derived from the hierarchy matrix. For example, the probability that *t*_1_ produces the best value is the sum of probabilities of the hierarchies {*t*_1_, *t*_2_, *t*_3_} and {*t*_1_, *t*_3_, *t*_2_} which is 25%. Similarly, the SUCRAs or P-scores can also be derived from the hierarchy matrix. Consider for example the SUCRA (or P-score) for treatment *t*_1_ in the hypothetical triangular network of Fig. 1; it is defined as

$$\frac{P\left({\mu}_{t_1{t}_2}>0\right)+P\left({\mu}_{t_1{t}_3}>0\right)}{2}$$

which can be calculated from the hierarchy matrix as

$$\frac{p_{\left\{{t}_1,{t}_2,{t}_3\right\}}+{p}_{\left\{{t}_1,{t}_3,{t}_2\right\}}+{p}_{\left\{{t}_3,{t}_1,{t}_2\right\}}+{p}_{\left\{{t}_1,{t}_2,{t}_3\right\}}+{p}_{\left\{{t}_1,{t}_3,{t}_2\right\}}+{p}_{\left\{{t}_2,{t}_1,{t}_3\right\}}}{2}=0.5$$

## Results

### Network of treatments for chronic obstructive pulmonary disease

**Fig. 3:**
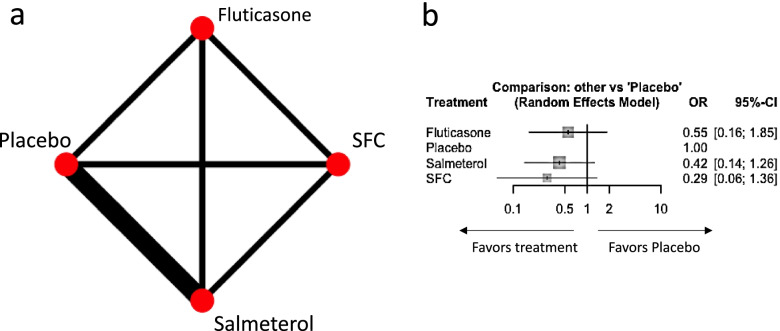
Network plot with edges proportional to the number of studies of each direct comparison (panel **a**) and network meta-analysis relative treatment effects (panel **b**) for a network of four treatments for chronic obstructive pulmonary disease. OR: odds ratio; CI: confidence interval; SFC: salmeterol fluticasone combination

We illustrate the process using a network comparing mortality rates in four treatments for chronic obstructive pulmonary disease: SFC (Salmeterol Fluticasone combination), Salmeterol, Fluticasone and Placebo [24]. Direct studies exist for all possible comparisons in the network, resulting in a fully connected network (Fig. 3 panel a). We synthesize data using the odds ratio as effect measure and assuming a random effects model and common heterogeneity variance across comparisons. SFC is associated with the greatest lowering in mortality compared to Placebo (odds ratio 0.29, 95% confidence interval 0.06 to 1.36), followed by Salmeterol (odds ratio 0.42, 95% confidence interval 0.14 to 1.26) and Fluticasone (odds ratio 0.55, 95% confidence interval 0.16 to 1.85) (Fig. 3 panel b). Heterogeneity standard deviation was estimated as $\hat{\tau}=0$ using a generalized method of moments estimate [25]. The hierarchy matrix of Table 2 was created using 10,000 simulations from the multivariate normal distribution with mean and variance covariance matrix being the respective quantities from the estimated NMA relative treatment effects. Table 2 lists all possible treatment hierarchies (*T* !  = 24) along with their frequency of occurring.

**Table 2:** Hierarchy matrix of the network of four treatments for chronic obstructive pulmonary disease of Fig. 3. Checks indicate the hierarchies that fulfil the criteria specified in the columns. Sum shows the probability of the criterion to hold. SFC: salmeterol fluticasone combination; Inf: infinity

Hierarchy	Frequency	Ratios	Criterion A Hierarchy is exactly ‘SFC, Salmeterol, Fluticasone, Placebo’	Criterion B Order “SFC, Fluticasone, Placebo” is retained	Criterion C Fluticasone is among the best two options	Criterion D SFC and Salmeterol are the two best options	Criterion E: Both criteria B AND C are satisfied
SFC, Salmeterol, Fluticasone, Placebo	28%	–	✓	✓		✓	
SFC, Fluticasone, Salmeterol, Placebo	19%	1.47		✓	✓		✓
Salmeterol, SFC, Fluticasone, Placebo	12%	2.33		✓		✓	
SFC, Salmeterol, Placebo, Fluticasone	9%	3.11				✓	
Salmeterol, Fluticasone, SFC, Placebo	7%	4.00			✓		
Fluticasone, SFC, Salmeterol, Placebo	6%	4.67			✓		
Fluticasone, Salmeterol, SFC, Placebo	5%	5.60			✓		
Salmeterol, SFC, Placebo, Fluticasone	4%	7.00				✓	
SFC, Fluticasone, Placebo, Salmeterol	3%	9.33		✓	✓		✓
Salmeterol, Fluticasone, Placebo, SFC	2%	14			✓		
Fluticasone, Salmeterol, Placebo, SFC	1%	28			✓		
SFC, Placebo, Salmeterol, Fluticasone	1%	28					
Fluticasone, SFC, Placebo, Salmeterol	1%	28			✓		
Salmeterol, Placebo, SFC, Fluticasone	1%	28					
Salmeterol, Placebo, Fluticasone, SFC	1%	28					
SFC, Placebo, Fluticasone, Salmeterol	0%	Inf					
Fluticasone, Placebo, Salmeterol, SFC	0%	Inf			✓		
Fluticasone, Placebo, SFC, Salmeterol	0%	Inf			✓		
Placebo, Salmeterol, Fluticasone, SFC	0%	Inf					
Placebo, SFC, Salmeterol, Fluticasone	0%	Inf					
Placebo, Salmeterol, SFC, Fluticasone	0%	Inf					
Placebo, Fluticasone, SFC, Salmeterol	0%	Inf			✓		
Placebo, SFC, Fluticasone, Salmeterol	0%	Inf					
Placebo, Fluticasone, Salmeterol, SFC	0%	Inf			✓		
Sum	100%	–	28%	62%	44%	53%	22%

We consider the following alternative hierarchy questions of interest as Step 1 of the suggested approach:

- Criterion A: Hierarchy is exactly ‘SFC, Salmeterol, Fluticasone, Placebo’
- Criterion B: Order ‘SFC, Fluticasone, Placebo’ is retained anywhere in the hierarchy
- Criterion C: Fluticasone is among the best two options
- Criterion D: SFC and Salmeterol are the two best options
- Criterion E: Both criteria B and C are satisfied

In Step 2 of the approach, we identify the hierarchies that satisfy the above criteria and in Step 3 we add their estimated probabilities (Table 2). Criterion A requires that an exact predefined hierarchy occurs and is thus fulfilled by a single hierarchy, ‘SFC, Salmeterol, Fluticasone, Placebo’, corresponding to the order of mean effects, which appeared with frequency 28%. The criterion that SFC is better than Fluticasone and Fluticasone is better than Placebo (criterion B) is satisfied by all hierarchies for which order ‘SFC, Fluticasone, Placebo’ is retained, with or without other treatments in between. Four hierarchies fulfill this criterion with frequencies 28, 19, 12 and 3%. Thus, the frequency for the particular order to be retained in the hierarchy is 62%. Half of the possible hierarchies should have Fluticasone ranked first or second as required by criterion C. The sum of the frequencies of the respective 12 hierarchies is 44%. Criterion D specifies SFC and Salmeterol in the first two ranks, which is satisfied in four hierarchies with a total frequency of 53%. For the combination criterion E to be satisfied, hierarchies where SFC is better than Fluticasone, Fluticasone is better than Placebo and Fluticasone ranks among the best two options are the target hierarchies; these hierarchies are two (‘SFC, Fluticasone, Salmeterol, Placebo’ and ‘SFC, Fluticasone, Placebo, Salmeterol’) with frequencies 19 and 3% respectively.

Consider that we are interested in evaluating the certainty of criterion A. The frequency of 28% for the most probable hierarchy can be judged by comparing it with that of the subsequent hierarchies. The probability ratios of the frequency of the most probable hierarchy with the second and the third hierarchies are 1.47 and 2.33 respectively (Table 2).

### Network of antidepressants for major depression

**Fig. 4:**
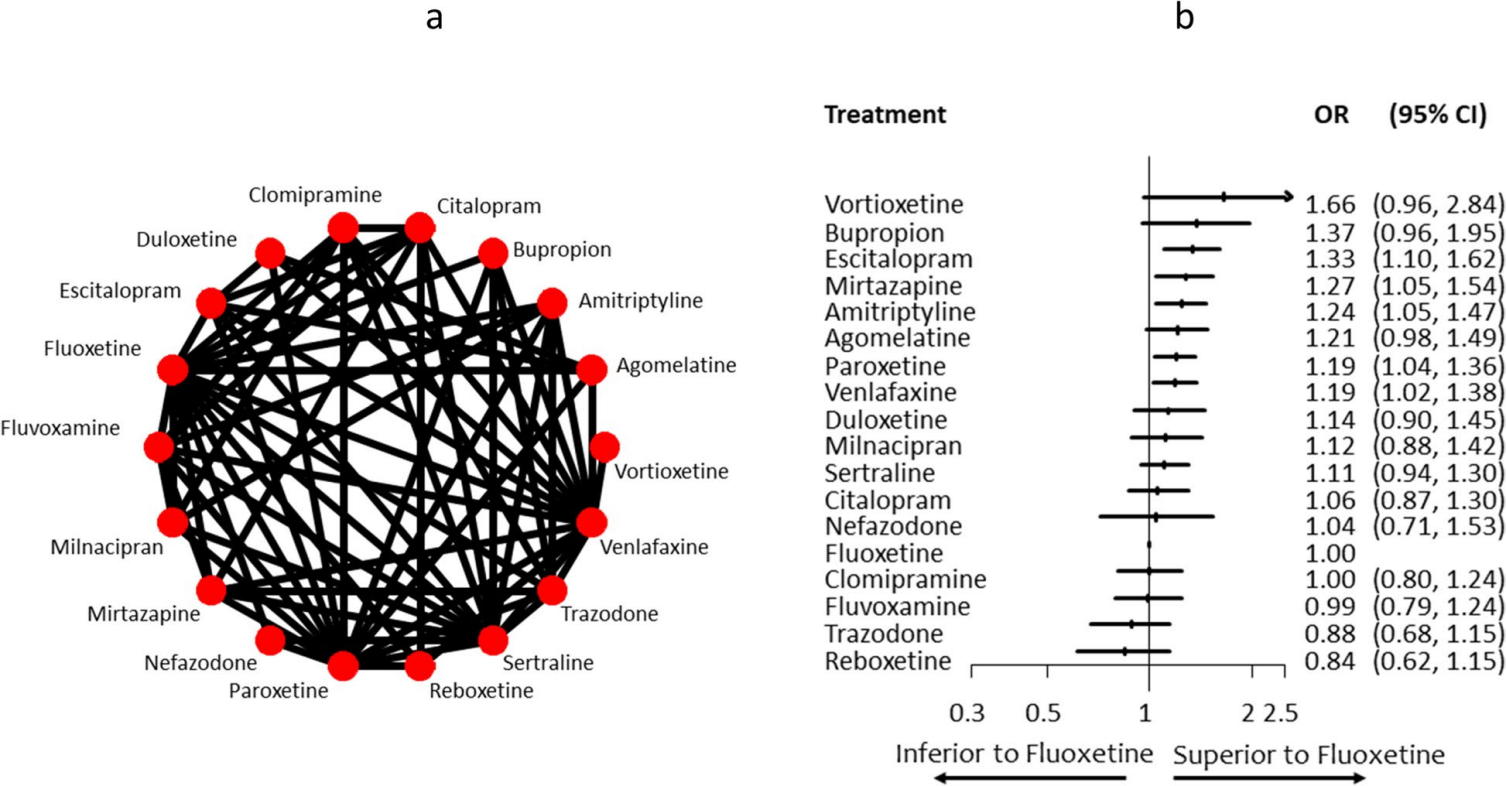
Network plot of head-to-head studies (panel **a**) and network meta-analysis relative treatment effects (panel **b**) for a network of 18 antidepressants major depression. OR: odds ratio; CI: confidence interval

To illustrate the methods in a larger network of interventions, we take a published NMA of 18 antidepressants for major depression, illustrated in Fig. 4 panel a [26]. We focus on the primary binary outcome ‘efficacy’, defined as at least 50% reduction in the symptoms’ scales between baseline and 8 weeks of follow up. Studies were synthesized using odds ratio and NMA relative treatment effects of all antidepressants versus Fluoxetine are shown in Fig. 4 panel b. Heterogeneity standard deviation was assumed common across comparisons and estimated as $\hat{\tau}=0.18$ using a generalized method of moments estimate [25]. The number of possible hierarchies is 18! rendering each hierarchy rare to occur. Out of the 500,000 simulations, only 7 hierarchies appeared twice, with the rest hierarchies appearing only once; in Table 3, the 7 most frequent hierarchies are listed.

**Table 3:** Most probable hierarchies for the network of antidepressants of Fig. 4

Vortioxetine, Bupropion, Escitalopram, Mirtazapine, Agomelatine, Amitriptyline, Duloxetine, Venlafaxine, Paroxetine, Citalopram, Milnacipran, Fluoxetine, Sertraline, Clomipramine, Fluvoxamine, Nefazodone, Trazodone, Reboxetine	
Vortioxetine, Bupropion, Escitalopram, Mirtazapine, Agomelatine, Milnacipran, Venlafaxine, Citalopram, Amitriptyline, Sertraline, Paroxetine, Duloxetine, Clomipramine, Fluvoxamine, Fluoxetine, Nefazodone, Trazodone, Reboxetine	
Vortioxetine, Bupropion, Escitalopram, Mirtazapine, Amitriptyline, Agomelatine, Duloxetine, Venlafaxine, Paroxetine, Milnacipran, Sertraline, Fluvoxamine, Citalopram, Fluoxetine, Clomipramine, Trazodone, Reboxetine, Nefazodone	
Bupropion, Vortioxetine, Escitalopram, Nefazodone, Mirtazapine, Agomelatine, Amitriptyline, Paroxetine, Milnacipran, Sertraline, Venlafaxine, Duloxetine, Fluvoxamine, Fluoxetine, Citalopram, Clomipramine, Trazodone, Reboxetine	
Vortioxetine, Bupropion, Mirtazapine, Escitalopram, Venlafaxine, Amitriptyline, Paroxetine, Agomelatine, Milnacipran, Citalopram, Sertraline, Clomipramine, Duloxetine, Nefazodone, Fluoxetine, Fluvoxamine, Trazodone, Reboxetine	
Bupropion, Vortioxetine, Escitalopram, Mirtazapine, Amitriptyline, Venlafaxine, Duloxetine, Sertraline, Agomelatine, Citalopram, Paroxetine, Clomipramine, Fluvoxamine, Milnacipran, Fluoxetine, Trazodone, Nefazodone, Reboxetine	
Vortioxetine, Bupropion, Escitalopram, Mirtazapine, Venlafaxine, Amitriptyline, Nefazodone, Sertraline, Agomelatine, Paroxetine, Citalopram, Clomipramine, Fluoxetine, Duloxetine, Milnacipran, Fluvoxamine, Trazodone, Reboxetine	

We consider the following alternative hierarchy questions of interest as Step 1 of the suggested approach:

- Criterion A: Vortioxetine ranks first, Bupropion second and Escitalopram third
- Criterion B: Vortioxetine, Bupropion and Escitalopram are the best three treatments
- Criterion C: Vortioxetine has better outcome value than that of Bupropion and Bupropion has better outcome value than that of Escitalopram
- Criterion D: Vortioxetine, Bupropion and Escitalopram have an odds ratio of 1.25 or higher against Fluoxetine
- Criterion E: Vortioxetine, Bupropion and Escitalopram have an odds ratio of 1 or higher against Fluoxetine

The estimated probability that Vortioxetine ranks first, Bupropion second and Escitalopram third (criterion A) is 9%. The estimated probability that these three treatments occupy any of the first three ranks (criterion B) is obviously higher; 19% of times Vortioxetine, Bupropion and Escitalopram were the three treatments with the highest odds ratios. The relative order of the first three treatments is also quite precise; in one third of simulations (33%), Vortioxetine performed better than Bupropion and Bupropion performed better than Escitalopram (criterion C).

Comparisons with a clinically important value are often more of interest than comparisons to the null effect. In the original NMA, an odds ratio of 0.8 and its reciprocal 1.25 was used for the examined outcome to judge upon imprecision of treatment effects and assess the confidence in NMA results. The estimated probability that all three treatments Vortioxetine, Bupropion and Escitalopram have an odds ratio of 1.25 or higher against the old, standard treatment Fluoxetine (criterion D) is 45%, while judging against the null (criterion E) the respective estimated probability is 92%.

## Discussion

In this paper, we suggest an approach to answer complex hierarchy questions in NMA and define the certainty around them. The approach that we took moves away from producing a plain, non-meaningful and difficult to interpret hierarchy and towards attaching treatment ranking to a clear decision question, relevant to all or a subset of competing treatments.

The work presented in this paper is somehow related to the precision in the treatment hierarchy from a NMA as a whole. Preliminary suggestions have associated the uncertainty of the entire treatment hierarchy with the shape of the rankograms: rankograms which show large differences between each treatment being at each rank indicate a precise treatment hierarchy, while flat rankograms reflect uncertainty in the treatment hierarchy [27]. This suggestion can be formalised with the use of the hierarchy matrix. A probability mass function where one hierarchy takes 100% probability and all others zero, has maximum possible precision. In contrast, when each of the *T*! possible hierarchies have probability 1/*T*!, the precision is the minimum possible. Precision can be reflected by the magnitude of ${p}_{h_1}$ (the closer to 1, the more precise the treatment hierarchy) or by summarizing the hierarchy matrix in various ways (e.g. taking the variance of all *h*_*l*_). Alternatively, the rate at which ratios of frequencies as those calculated in Table 2 increase also give an indication of the precision of the treatment hierarchy.

However, all these approaches suffer from the drawback that they are dependent on the number of treatments and thus cannot be used as a universal way to judge the precision of the entire treatment hierarchy. In contrast, looking at the certainty of the specified criteria of interest to hold is more meaningful and relevant and constitutes an alternative way of judging imprecision of NMA treatment effects and treatment ranking. Even examples with relatively imprecise results (reflected in wide, overlapping confidence intervals) may be associated with considerable certainty around specific criteria, relevant for decision making. Thus, carefully choosing criteria based on which to judge the imprecision of NMA results is particularly important.

### Limitations

The proposed method has several limitations. In decision making multiple outcomes are often of interest and the current approach cannot take this into consideration. Moreover, benefit-risk assessments between different outcomes may be of interest to decision makers but cannot currently be appropriately handled. It would be potentially helpful that the derived frequencies are accompanied by 95% confidence intervals; as, however, the interpretation of such intervals would be unclear, they are not incorporated in the current version of the **nmarank** package [21].

### Future directions

The method presented in this paper can be extended to adapt to decision questions related to more than one outcome. For example, we could sample from two or more outcomes and measure the frequency for each combination of hierarchies for the considered outcomes. Taking for example a network with two outcomes, O1 and O2 we would calculate ${p}_{h_l,O1}\cap {p}_{h_l,O2}$. The two outcomes can be sampled either separately or simultaneously, calculating or imputing within and between outcomes correlation, as described elsewhere [28, 29]. As all combinations of hierarchies for two (or more) outcomes are to be considered, estimated probabilities for each specific combination will be smaller compared to those from a single outcome.

## Conclusions

Medical societies, national and international agencies, guideline panels and clinicians very often use evidence synthesis to make informed decisions and recommendations about the clinical effectiveness of alternative treatment options [30]. Given the need to make treatment recommendations, producing a hierarchy is natural within the aims of NMA end-users. We recommend that treatment hierarchies are attached to a specific decision question, a practice which is currently rarely undertaken in NMA applications. Depending on the setting, estimated probabilities of set criteria might inform or guide decision making regarding the choice of the preferable treatments and modify accordingly clinical practice. In conclusion, the method described in this paper offers an approach to produce clinically relevant output from NMA, which is specifically related to the research question of the particular systematic review.

## Data Availability

Outcome data and the code for applying our methods are available in https://github.com/esm-ispm-unibe-ch/nmarank/tree/reproducible.

## Abbreviations

NMA: Network Meta-Analysis
SUCRA: Surface Under the Cumulative RAnking curve
